# Soil microbial C:N ratio is a robust indicator of soil productivity for paddy fields

**DOI:** 10.1038/srep35266

**Published:** 2016-10-14

**Authors:** Yong Li, Jinshui Wu, Jianlin Shen, Shoulong Liu, Cong Wang, Dan Chen, Tieping Huang, Jiabao Zhang

**Affiliations:** 1 Changsha Research Station for Agricultural & Environmental Monitoring and Key Laboratory of Agro-ecological Processes in Subtropical Regions, Institute of Subtropical Agriculture, Chinese Academy of Sciences, Hunan 410125, China; 2 Faculty of Veterinary and Agricultural Sciences, The University of Melbourne, Victoria 3010, Australia; 3 Hunan Soil and Fertiliser Station, Hunan 410005, China; 4 Institute of Soil Science, Chinese Academy of Sciences, Nanjing, Jiangsu 210008, China

## Abstract

Maintaining good soil productivity in rice paddies is important for global food security. Numerous methods have been developed to evaluate paddy soil productivity (PSP), most based on soil physiochemical properties and relatively few on biological indices. Here, we used a long-term dataset from experiments on paddy fields at eight county sites and a short-term dataset from a single field experiment in southern China, and aimed at quantifying relationships between PSP and the ratios of carbon (C) to nutrients (N and P) in soil microbial biomass (SMB). In the long-term dataset, SMB variables generally showed stronger correlations with the relative PSP (rPSP) compared to soil chemical properties. Both correlation and variation partitioning analyses suggested that SMB N, P and C:N ratio were good predictors of rPSP. In the short-term dataset, we found a significant, negative correlation of annual rice yield with SMB C:N (r = −0.99), confirming SMB C:N as a robust indicator for PSP. In treatments of the short-term experiment, soil amendment with biochar lowered SMB C:N and improved PSP, while incorporation of rice straw increased SMB C:N and reduced PSP. We conclude that SMB C:N ratio does not only indicate PSP but also helps to identify management practices that improve PSP.

Paddy soils are important for food security in the world, as they take up an area of 123 million hectares, and are responsible for providing staple food for more than half of the world population^1^,^2^. Among the areas of paddy soils, 25% are in China and produce grain that feed more than 60% of the total Chinese population^2^. Since 1980 s, apart from encouraging large amounts of nitrogen fertilisers, the Chinese government has promoted the agricultural practices of legume rotation, farmyard manure amendment and straw incorporation to sustainably increase paddy soil productivity (PSP)^3^. In recent years, rice straw incorporation has become mandatory because the conventional practice of burning rice straw is banned by law to improve regional air and soil quality, while legume rotation and manure additions are almost dying out in paddy fields because farmers are becoming less willing to carry them out. Despite the contribution of new rice hybrids, PSP is declining gradually^4^.

Finding an appropriate method for assessing PSP remains a challenge. Numerous methods have been developed to evaluate soil productivity using physical, chemical and biological characteristics of paddy soils^4^,^5^,^6^,^7^,^8^,^9^,^10^,^11^, with most focusing on soil physical and chemical properties^12^. Bastida *et al*. pointed out that the structural and functional relationships of soil microbial communities are considered as probable future indicators for soil quality^5^. In recent years, phospholipid fatty acid (PLFA), denaturing gradient gel electrophoresis (DGGE) and high-throughput sequencing analysis have been increasingly deployed to investigate the abundance and composition of soil microbial groups^13^,^14^,^15^,^16^,^17^. For example, Frostegård *et al*.^18^ used the PLFA method to investigate the microbial communities from two soil types experimentally exposed to different heavy metals. Muyzer *et al*.^19^ applied DGGE to analyze the microbial community structure of sea sediment. Roesch *et al*.^20^ used high throughput DNA pyrosequencing to assess the bacterial diversity in four soils across a large transect of the western hemisphere. Trivedi *et al*.^21^ compiled a global dataset from both published and unpublished data of soil bacterial diversity and composition evaluated with next generation sequencing techniques; their meta-analysis suggested that the microbial indicators (e.g., relative abundance of certain bacterial phyla: *Actinobacteria* and *Chloroflexi*) can be developed as tools to predict primary productivity and soil health.

Soil microbial biomass (SMB) is widely regarded as an early indicator of changes in soil quality resulting from land management in ecosystems^22^,^23^,^24^,^25^. The soil microbial biomass carbon (SMB C), nitrogen (SMB N) and phosphorus (SMB P) constitute 1–7% of soil organic carbon (SOC), 1–5% of total soil nitrogen (TSN) and 1–5% of total soil phosphorus (TSP), respectively^26^,^27^,^28^,^29^,^30^,^31^. They are amongst the most labile C, N and P pools in soils^32^. They also act as the sink or the source of nutrients available for crop uptake, and thus play important roles in forming soil productivity of agroecosystems. Usually, the PSP (expressed in rice yield) correlates positively with SMB C, SMB N or SMB P^4^,^11^,^33^,^34^, but sometimes does not^35^,^36^,^37^. It is thus possible that the individual elemental amounts of soil microbial biomass may not ultimately determine PSP.

Insights into stoichiometry of the main nutrient elements in the soil and the soil microbial biomass can unveil key ecological processes of ecosystems at spatial scales from soil aggregates to continents^38^,^39^,^40^, and as a result these elemental ratios can influence ecosystem structure, species composition and diversity, ecosystem functions and provision of ecosystem services, including soil productivity^41^,^42^,^43^. The C:nutrient ratio is a fundamental indicator of biogeochemical cycles in ecosystems^44^. Any shift in C:nutrient stoichiometry could pose great impacts on the nutrient cycling and the composition and structure of plant communities, affecting ecosystem service functions at local, regional, national and global scales^45^. The relationships between soil productivity and SMB C:nutrient ratios in agroecosystems are not well explored. Besides positive correlations of grain yields with SMB C and SMB N, Yusuf *et al*. reported a negative correlation of grain yield with SMB C:N ratio in a maize-legume dryland cropping system in Nigeria^46^; such a relationship has not yet been reported in paddy soils. Unravelling the relationship between SMB C:nutrient ratios and soil productivity in paddy agroecosystem may not only help us develop more reliable biological indicators for evaluating soil productivity but also improve decision making for applying appropriate agricultural practices to increase soil productivity and sustainability.

The aim of this research was to investigate the quantitative relationship between soil productivity and SBM C:nutrient ratios in paddy fields. Firstly, we used a long-term (1987–1999) dataset from paddy fertilisation experiments, carried out in eight counties of Hunan Province, China to develop such a relationship. These experiments, with a double rice cropping system, had five treatments: CK - non fertiliser application, NPK - 100% chemical fertiliser application, OM3 - 70% of applied N fertiliser in chemical form and 30% in manure, OM6 - 40% of applied N fertiliser in chemical form and 60% in manure, and CON - local farmers’ practices with most of the applied N in chemical form and the rest in manure (see Supplementary Tables S1–S4 and Fig. S1). Then, we examined a short-term (2012–2014) dataset of a paddy fertilisation experiment carried out at Jinjing in Changsha County of Hunan Province to verify the quantitative relationship developed from the long-term dataset. This experiment was also managed with a double rice cropping system and had eight treatments: NPK - 100% chemical fertiliser application, NPK + LS - 100% chemical fertiliser plus 3 Mg DM ha^−1^ of rice straw, NPK + HS - 100% chemical fertiliser plus 6 Mg DM ha^−1^ of rice straw, NPK + LC - 100% chemical fertiliser plus 24 Mg DM ha^−1^ of biochar, NPK + HC - 100% chemical fertiliser plus 48 Mg DM ha^−1^ of biochar, 0.5NPK + PM - 50% N in chemical fertiliser and the rest in pig manure, NPK + F - 100% chemical fertiliser application with continuous flooding, and NPK + HS + F - 100% chemical fertiliser application plus 6 Mg DM ha^−1^ of rice straw with continuous flooding (see Supplementary Tables S5 and S6).

## Results

**Figure 1:**
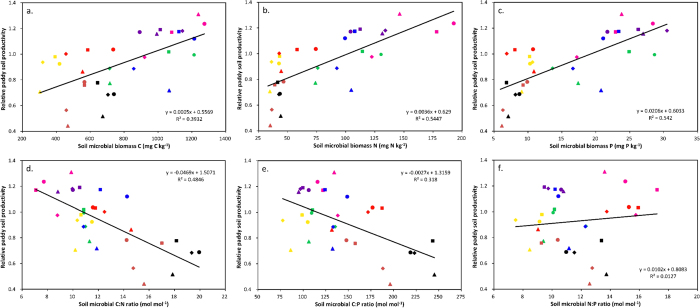
Relationships of relative paddy soil productivity with SMB (**a**) C, (**b**) N, (**c**) P, (**d**) C:N ratio, (**e**) C:P ratio and (**f**) N:P ratio of four treatments (represented by four symbols: ▲ – CON, ♦ – NPK, ■ – OM3 and ● – OM6) at eight county sites (represented by eight colors: 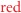 Changsha, black – Hanshou, 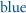 – Linli, 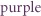 – Nanxian, 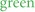 – Ningxiang, 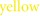 – Xinhua, 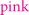 – Wugang and 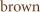 – Zhuzhou) in the long-term dataset.

**Figure 2:**
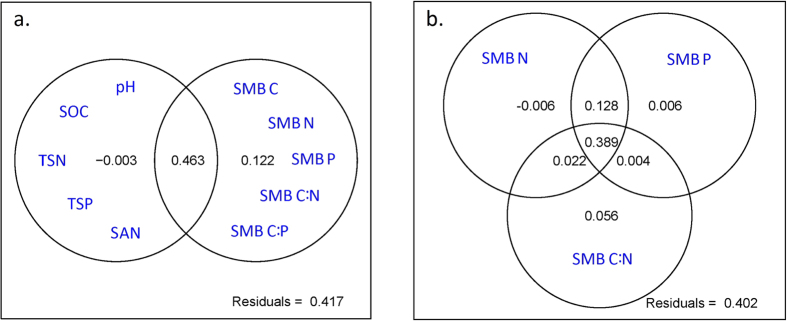
Venn diagrams describing the partitioning of the variation of relative paddy soil productivity among two types of three sets of explanatory variables: (**a**) soil chemical properties [pH, SOC, TSN, TSP and SAN] and soil biological properties [SMB C, SMB N, SMB P, SMB C:N and SMB C:P], and (**b**) SMB N, SMB P and SMB C:N, in the long-term dataset. The bounding rectangle represents the total variation in relative paddy soil productivity, while each circle represents the portion of variation accounted for by an explanatory variable or a combination of explanatory variables.

For the long-term dataset observed at eight county sites, the relative paddy soil productivity (rPSP, the ratio of the average annual rice yield increase for 1987–1999 for a given treatment to the average annual rice yield for 1987–1999 for the control treatment) showed varied correlations with soil chemical and biological properties (see Table 1). The chemical properties pH, SOC, TSN, TSP and soil available N (SAN) exhibited significant, positive correlations with rPSP (*P* < 0.01), while no correlations were present between rPSP and soil C:nutrient ratios (*P* = −0.24–0.05). The correlations of rPSP with microbial variables were promising (Table 1 & Fig. 1). The result of variation partitioning analysis (VPA), as shown in Fig. 2a, indicated that 58.3% of the variation of rPSP were explained by soil chemical (pH, SOC, TSN, TSP and SAN) and biological (SMB C, SMB N, SMB P, SMB C:N and SMB C:P) properties. In addition, the soil chemical and biological properties independently explained −0.3% and 12.2% of the variation of rPSP, respectively, and they shared a large proportion of the variation (46.3%). Except for the SMB N:P ratio, all SMB variables showed significant correlations with rPSP (*P* < 0.001), and the highest correlations were found with SMB N (*r* = 0.74), P (*r* = 0.74) and C:N ratio (*r* = −0.70). As presented in Table 1, the SMB C, N and P were highly inter-correlated (*r* = 0.88–0.91), and the ratios of SMB C:N and C:P were also highly correlated (*r* = 0.81). SMB C:N ratio showed significant correlations with SMB N and P (*r* = 0.64–0.68) and a more evenly-distributed relationship with rPSP in Fig. 1d than SMB N and P did in Fig. 1b,c. The result of the second VPA to assess the contributions of SMB N, P and C:N ratio to the variation of rPSP was shown in Fig. 2b. Despite of a large amount of intersection (38.9%) among the three biological variables, SMB C:N ratio still independently explained 5.6% of the variation of rPSP, much more than by SMB N (−0.6%) and P (0.6%).

**Figure 3:**
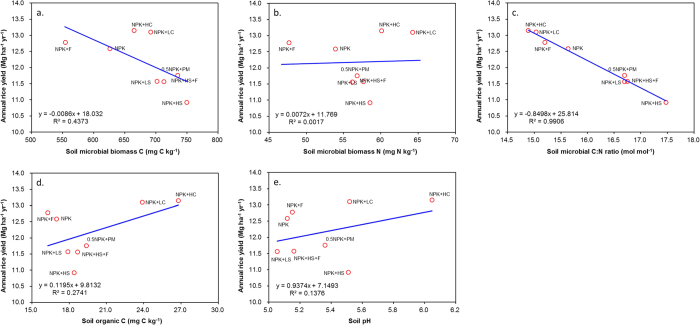
Relationships of paddy soil productivity (represented by annual rice yield) with SMB (**a**) C, (**b**) N, (**c**) C:N ratio, (**d**) SOC and (**e**) soil pH of eight fertilisation treatments at Jinjing in Changsha County in the short-term dataset.

For the short-term dataset observed at Jinjing in Changsha County, as seen in Fig. 3, we observed a significant, negative correlation of average annual rice yield with SMB C:N ratio (*r* = −0.99, *P* ≈ 0), but no correlations with SMB C and N, SOC and pH (*P* > 0.05)

## Discussion

For the long-term dataset, the chemical properties pH, SOC, TSN, TSP and SAN exhibited significant, positive correlations with rPSP (*P* < 0.01), while no correlations were presented between rPSP and soil C:nutrient ratios (*P* = −0.24–0.05) (see Table 1). These findings confirm previous studies^4^,^11^. For the soil biological properties, except for the SMB N:P ratio, all SMB variables showed significant correlations with rPSP (|*r*| = 0.56–0.74, *P* < 0.001), clearly indicating a link between higher rice yields and higher microbial activity. As shown in Fig. 2a, the soil biological properties independently explained 12.2% of the variation of rPSP, suggesting greater impact of soil biological properties on rPSP compared to soil chemical properties.

The significant, negative correlations between rPSP and ratios of SMB C:N (*r* = −0.70) and C:P (*r* = −0.56) shown in Fig. 1d,e were interesting as they were previously unreported for paddy soils. These results can be explained by that a high SMB C:N or C:P ratio (resulted from either the added organic materials with high C:N or C:P ratio, the low input of N or P fertiliser, or the intrinsically high soil C:N or C:P ratio) contributes to substantial N or P immobilization, which reduces the flux of mineral N or P in paddy soils, and is thus detrimental to rice yield^46^,^47^. Based on the fact that they presented the highest correlations with rPSP in this long-term dataset (|*r*| = 0.70–0.74), we propose SMB N, P and C:N ratio as the soil biological properties that best indicate the soil productivity of paddy fields. The second VPA test (see Fig. 2b) to assess the contributions of SMB N, P and C:N ratio to the variation of rPSP drove us to contend that the SMB C:N ratio is a more robust indicator of PSP than SMB N and SMB P. The linear regression relationship (*y* = −0.0469*x* + 1.51) presented in Fig. 1d also illustrated that a unit reduction in SMB C:N ratio increased rPSP by approximately 4.5%. It should be noted that this relationship was derived from a large range of SMB C:N ratios. Therefore, the correlation between SMB C:N ratio and rPSP may not hold true for the data from the experiments in Wugang, Xinhua and Nanxian, where the differences in SMB C:N ratios among the treatment were small.

As seen in Fig. 3, we observed a significant, negative correlation of average annual rice yield with SMB C:N ratio (*r* = −0.99, *P* ≈ 0), but no correlations with SMB C and SMB N (*P* > 0.05), in the short-term dataset. Confirming the finding from the long-term dataset, the SMB C:N ratio remained an effective indicator of PSP. Given the fact that there were nine measurements of SMB for each treatment in the short-term dataset from 2012 to 2014 and only one for the long-term dataset in 2001, the relationship between PSP and SMB C:N ratio observed in the short-term dataset was more credible. Based on the linear regression relationship (*y* = −0.850*x* + 25.8) shown in Fig. 3c, a unit reduction in SMB C:N ratio resulted in an increase of 0.85 Mg ha^−1^ yr^−1^ in PSP. However, we have to read the relationship in Fig. 3c cautiously, as the SMB variables in the short-term dataset only sat at either the low or the high end in the long-term dataset (see Fig. 1a,b,d). The lack of correlation between SMB N and PSP in the short-term dataset may be due to low SMB N (all were less than 65 mg N kg^−1^). As shown in Fig. 1b for the long-term dataset, when SMB N was lower than 50 mg N kg^−1^, the correlation between SMB N and rPSP became insignificant. To definitively confirm the relationships between PSP and SMB C:nutrient stoichiometry, a full-scale verification dataset in terms of the measurements of SMB variables is needed in the future.

For the soil chemical properties, the correlation analysis showed that rice yield did not have significant correlations with SOC and soil pH in the short-term dataset (Fig. 3d,e). Combined with the same correlations presented in Table 1 for the long-term dataset, the routinely measured soil chemical properties (e.g., SOC and pH) may not predict PSP as accurately as SMB C:N ratio. Although the determination of SMB C, N and P by the fumigation-extraction method^48^,^49^ is relatively complicated, time-consuming and costly, the soil microbial variables advanced the routine soil chemical properties (e.g., SOC, pH, TSN, TSP, SAN and SAP) as the indicator of PSP because most of the important fluxes of C, N and P in paddy soils (e.g., mineralization/immobilization, nitrification/denitrification and biological N fixation) are controlled by soil microorganisms^50^.

Other types of soil biological properties may indicate PSP. The ratio of fungi to bacteria (F:B ratio) in the soil determined by the PLFA method can be used to indicate soil quality, and it tends to increase with the soil C:N ratio^51^. However, the quantitative relationship of F:B ratio with soil productivity has not yet been investigated. Theoretically, the SMB C:N ratio is associated with, but not equivalent to, the F:B ratio. This is because fungi-dominant soils tend to have high SMB C:N ratios due to the fact that the C:N ratios in fungi (8:1–29:1) are greater than in bacteria (4:1–8:1)^52^. The F:B ratio is often converted into a F:B biomass C ratio using a prescribed conversion factor such as 27.4 suggested by Klamer & Bååth^53^, Joergensen & Wichern^54^ and Bezemer *et al*.^55^. Thus, the SMB C:N ratio and the F:B ratio are not closely related, and do not explain each other. Most recently, the relative abundance of several bacterial phyla (e.g., *Actinobacteria* and *Chloroflexi*) determined by next generation sequencing techniques in agricultural systems across the globe is found to be a potential indicator for assessing primary productivity and soil health^21^. Therefore, the quantitative relationship between the abundance of these selected bacterial phyla and PSP needs to be investigated.

The relationship between PSP and SMB C:N ratio is also helpful in evaluating the effectiveness of land management practices in improving crop yields. As shown in Fig. 1d for the long-term dataset, the treatments with manure amendments (OM3 and OM6) tended to improve PSP more than the CON and NPK treatments. For the short-term dataset, the relationship between PSP and SMB C:N ratio (see Fig. 3c) showed that: i) rice straw incorporation in paddy fields actually did not improve soil productivity and decreased soil productivity at high rates of straw application because the incorporated straw drove up SMB C:N ratio, ii) the practice of continuous flooding slightly improved PSP, and iii) the amendment of biochar in paddy fields was the only measure that improved PSP compared to the 100% chemical fertiliser treatment.

A number of studies reported that biochar addition in soils can increase SMB, and may also affect the soil biological community composition, which in turn affects nutrient cycling, plant growth, greenhouse gas emissions and SOC mineralization^56^,^57^. Our short-term dataset indicated that biochar application increased SMB and rice yield. In addition, the biochar-priming effect on SMB N was stronger than SMB C, and as a result the amendment of biochar reduced SMB C:N ratio in paddy fields (see Supplementary Table S6), in contrast to the finding in a wheat-cropped dryland soil in the North China Plain where biochar application increased SMB C:N ratio^58^. The mechanism underpinning the reduction of SMB C:N ratio induced by the application of biochar in paddy fields is unclear. Yusuf *et al*. reported a significant decrease of SMB C:N ratio when legumes were introduced in a dryland maize-cropping system^46^, which suggests that there was possibly a N enrichment process such as biological N fixation primed in the extremely porous structure of pyrolyzed biochar in our biochar-amended paddy fields. Chen *et al*. have recently reported that biochar addition in rice cropping systems led to a significant increase of N-fixing bacteria *Bradyrhizobium* in three paddy soils in southern China^59^. Further research is needed to identify such processes.

In summary, based on our long-term and short-term datasets, the elemental stoichiometric indicator of SMB C:N ratio was found to be a robust predictor of PSP in southern China. Such a soil biological index can not only serve as an effective indicator of PSP, but also help us to identify management practices that improve PSP. As our results were based on experimental sites located at eight counties in Hunan Province, more paddy soil samples need to be analyzed in other regions to verify the correlation between SMB C:N ratio and PSP found in this study. Also, microbial abundance and composition need to be analyzed to investigate microbial mechanisms among soils with different SMB C:N ratios. Though yet un-validated, SMB C:N ratio may apply to other cropping systems as an effective indicator for soil productivity because it is closely related to the microbial-mediated C, N and P fluxes in soils. Moreover, to incorporate the SMB C:N ratio test into a decision support system for paddy soil management, a comprehensive study should be carried out to develop base-line values and ranges of SMB C:N ratio for various soil types and climate regions.

## Methods

### Date compilation

In this study, two datasets of paddy fertilisation experiments, one long-term (1987–1999) and one short-term (2012–2014), were used to investigate the quantitative relationships between PSP and SMB C: nutrient ratios in rice paddies.

The long-term paddy fertilisation experiments were conducted from 1987 to 1999 in typical paddy fields at eight county sites (Changsha - CS, Hanshou - HS, Linli - LL, Nanxian - NA, Ningxiang - NX, Wugang - WG, Xinhua - XH, Zhuzhou - ZZ) in Hunan Province, China. The climate, cropping system and basic soil properties of the 0–20 cm depth at the eight county experimental sites were described in Supplementary Table S1. The Ordination plot of the experimental sites using the chemical properties of the sites was shown in Supplementary Fig. S1, suggesting that the soil characteristics of the eight county experimental sites can be illustrated by using the principal components analysis. The first principal component (x-axis) showed high loadings on SOC and TSN, while the second principal component (y-axis) showed high loadings on soil pH and TSP. Thus, the experimental sites can then be grouped into four types. The first type had high SOC and TSN contents and relative low pH (e.g., ZZ and LL); the second type has high SOC, TSN and pH (e.g., NA, WG); the third type had relative low SOC and TSN contents and low pH (e.g., HS, NX); and the fourth type had low SOC and TSN contents (e.g., CS and XH). At each county experimental site, five treatments were commenced: CK – non-fertilised, NPK - 100% chemical fertiliser application, OM3 - 70% of applied N fertiliser in chemical form (urea) and 30% in manure, OM6 - 40% of applied N fertiliser in chemical form (urea) and 60% in manure, and CON - local farmers’ practices with most of applied N fertiliser in chemical form (urea) and the rest in manure. Detailed annual fertiliser application rates for N, P and potassium (K) at each experimental site were listed in Supplementary Table S2. These fertiliser application rates for the treatments of NPK, OM3 and OM6 were determined by considering soil indigenous nutrient supply and crop nutrient demand; therefore, they were different for each of the experimental sites. The average yields of individual early rice and late rice at each county site for 1987–1999 were summarised in Supplementary Table S3. To delineate the influence of intrinsic or basic soil fertility and parental materials on PSP at different county sites, a term of relative PSP, rPSP, was proposed. In this study, the rPSP for a given county site was defined by the following equation:


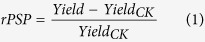


where *Yield* and *Yield*_*CK*_ are the average annual rice yields for 1987–1999 (Mg ha^−1^ yr^−1^) of any given fertilisation treatment and the non-fertilised treatment, respectively. The rice yield was measured by the quadrant method.

For the long-term dataset, soil samples were collected at a depth of 0–20 cm in 2001 after late rice harvest. Each soil sample consisted of a homogenized mixture of 8–10 soil cores collected in an “S” type in each experimental plot. The SOC content was determined through a wet digestion method using potassium dichromate^60^. The TSN content of the samples was determined using an automated C/N analyzer, while the TSP content was determined via a colorimetric method^61^ using an alkaline oxidation digestion procedure^62^. The SAN content was determined by extracting soil samples using 0.5 M K_2_SO_4_ with the extracts analyzed on a flow-injection auto-analyzer for NH_4_^+^-N and NO_3_^−^-N (Foss, Sweden). The soil available P (SAP) content was measured by extracting soil with 0.5 M NaHCO_3_ at pH 8.5^63^. The SMB C and N contents were measured using the chloroform (CHCl_3_) FE method^48^,^49^. For these measurements, three portions of pre-incubated soil (25 g on an oven-dried basis) were placed in a vacuum desiccator and exposed to alcohol-free CHCl_3_ vapor at room temperature for 24 h. The samples were then transferred to a clean desiccator, and residual CHCl_3_ was removed by evacuation for 20 min. The fumigated portions, together with the equivalent non-fumigated portions, were extracted with 80 ml of 0.5 M K_2_SO_4_; 15 ml of the 0.5 M K_2_SO_4_ extracts were then used to analyse the organic C contents in an automated carbon analyser (Phoenix 8000, USA), and 20 ml of the extracts were subjected to the analysis of total N after digestion in a flow injection analyzer (Foss, Sweden). Using a universal conversion factor of 0.45, we calculated the amounts of SMB C and SMB N according to the increase in extractable C and N in the fumigated soil compared to the control^49^. SMB P was determined according to Brookes *et al*.^64^. With the exception that a 4.0 g soil sample (on an oven-dried basis) was used, the fumigation procedure was the same as for SMB C and SMB N. The fumigated and non-fumigated portions were both extracted with 0.5 M NaHCO_3_. The 0.5 M NaHCO_3_ extracts were then analysed to determine total P using a colorimetric method^61^. At the same time, the recovery of P during the extraction was measured by adding a spike of inorganic P. Using a conversion factor of 0.40 and the recovery of an inorganic P spike, SMB P was calculated through measurement of the increase in extractable P in the fumigated soil compared to the extractable P in the control^64^. The measured soil nutrient contents for the long-term experiment were summarised in Supplementary Table S4.

The short-term paddy fertiliser experiment was conducted during 2012 to 2014 in a typical double rice field at Jinjing in Changsha County of Hunan Province. The climate, cropping system and basic soil properties of the 0–20 cm depth for the experimental site were described in Supplementary Table S5. There were eight treatments in this experiment: NPK - 100% chemical fertiliser application, NPK + LS - 100% chemical fertiliser plus 3 Mg DM ha^−1^ of rice straw, NPK + HS - 100% chemical fertiliser plus 6 Mg DM ha^−1^ of rice straw, NPK + LC - 100% chemical fertiliser plus 24 Mg DM ha^−1^ of biochar, NPK + HC - 100% chemical fertiliser plus 48 Mg DM ha^−1^ of biochar, 0.5NPK + PM - 50% N in chemical fertiliser and the rest in pig manure, NPK + F - 100% chemical fertiliser application with continuous flooding, and NPK + HS + F - 100% chemical fertiliser application plus 6 Mg DM ha^−1^ of rice straw with continuous flooding. The total fertiliser application rates for N (urea), P (calcium superphosphate) and K (KCl) were 120 kg N ha^−1^, 17.5 kg P ha^−1^ and 43.7 kg K ha^−1^, respectively, in the early rice season and 150 kg N ha^−1^, 17.5 kg P ha^−1^ and 43.7 kg K ha^−1^, respectively, in the late rice season. The water regime for the treatments other than NPK + F and NPK + HS + F was intermittent irrigation. For the water regime of intermittent flooding, after rice seedling transplanting, the paddy fields remained flooded for approximately 30 days. Then, a 10-day mid-season drainage was imposed, followed by intermittent irrigations until one week before rice harvest. For the water regime of continuous flooding, the difference between it and the intermittent flooding is that there is not mid-season drainage conducted, and the rice paddy was continuously flooded until one-week before harvest. The experiment was set up by a randomized block design with three replicates, and each field plot had an area of 35 m^2^ (7 m × 5 m). The rice cultivars used in this trial were Xiangzaoxian 45 for early rice and T-you 207 for late rice. Soil pH and SOC of soil samples of 0–20 cm for each treatment were determined once after late rice harvest each year, while SMB C and N were measured three times each year in spring, summer and autumn. The average annual grain yields and the measured mean values of SMB C, SMB N and SMB C:N ratio for the depth of 0–20 cm during 2012–2014 were illustrated in Supplementary Table S6. The measurement methods for SMB C and N, pH and SOC were the same as those used in the long-term experiments. The rice yield was also measured by the quadrant method.

### Data analysis

All statistical data analyses were performed using R software (http://www.r-project.org). Microsoft Excel 2010 and R software were used to produce the graphs. At first, the Pearson’s correlation analysis and the linear regression analysis were used to evaluate the relationships between PSP and soil properties, including SMB variables. Then, the variation partitioning^65^ analysis (VPA) was performed to attribute the variation observed in PSP to the significant soil properties to identify the best set of predictors of PSP. VPA was performed by the function of “varpart” in the “vegan” package in R software, which can partition variation among up to four variables (or groups of variables). Note that “varpart” is based on redundancy analysis and uses adjusted R^2^ to express explained variation.

## Additional Information

**How to cite this article**: Li, Y. *et al*. Soil microbial C:N ratio is a robust indicator of soil productivity for paddy fields. *Sci. Rep*. **6**, 35266; doi: 10.1038/srep35266 (2016).

## Supplementary Material

Supplementary Information

Supplementary Data

## Figures and Tables

**Table 1:** Matrix of Pearson’s correlation coefficients of relative paddy soil productivity (rPSP) with soil chemical and biological properties of four treatments at eight county sites in the long-term dataset.

	pH	SOC	TSN	TSP	C:N	C:P	N:P	SMB C	SMB N	SMB P	SMB C:N	SMB C:P	SMB N:P	SAN	SAP	rPSP
pH	—															
SOC	0.62***	—														
TSN	0.75***	0.93***	—													
TSP	0.51**	0.58***	0.61***	—												
C:N	−0.33	−0.06	−0.42*	−0.24	—											
C:P	0.12	0.48**	0.37*	−0.41*	0.21	—										
N:P	0.32	0.49**	0.57***	−0.27	−0.31	0.86***	—									
SMB C	0.62***	0.58***	0.77***	0.56***	−0.61***	0.05	0.38*	—								
SMB N	0.74***	0.69***	0.83***	0.55**	−0.46**	0.15	0.40*	0.89***	—							
SMB P	0.75***	0.63***	0.74***	0.71***	−0.36*	−0.05	0.15	0.88***	0.91***	—						
SMB C:N	−0.60***	−0.37*	−0.38*	−0.43*	−0.03	0.12	0.11	−0.36*	−0.68***	−0.64***	—					
SMB C:P	−0.46**	−0.28	−0.19	−0.44*	−0.32	0.20	0.34	−0.22	−0.44*	−0.61***	0.81***	—				
SMB N:P	0.11	0.15	0.32	−0.11	−0.52**	0.25	0.51**	0.23	0.33	−0.06	−0.11	0.47**	—			
SAN	0.55***	0.77***	0.90***	0.59***	−0.58***	0.26	0.55***	0.83***	0.72***	0.68***	−0.09	0.06	0.30	—		
SAP	−0.08	0.19	0.14	0.71***	0.00	−0.46**	−0.45**	0.27	0.18	0.34	−0.11	−0.24	−0.24	0.25	—	
rPSP	0.67***	0.46**	0.55**	0.52**	−0.24	−0.08	0.05	0.63***	0.74***	0.74***	−0.70***	−0.56***	0.11	0.38*	0.13	—

Note: pH - soil pH value, SOC - soil organic C content (g C kg^−1^ soil), TSN - total soil N content (g N kg^−1^ soil), TSP - total soil P content (g P kg^−1^ soil), C:N - soil C:N ratio (mol mol^−1^), C:P - soil C:P ratio (mol mol^−1^), N:P - soil N:P ratio (mol mol^−1^), SMB C - soil microbial biomass C amount (mg C kg^−1^ soil), SMB N - soil microbial biomass N amount (mg N kg^−1^ soil), SMB P - soil microbial biomass P amount (mg P kg^−1^ soil), SMB C:N - soil microbial C:N ratio (mol mol^−1^), SMB C:P - soil microbial C:P ratio (mol mol^−1^), SMB N:P - soil microbial N:P ratio (mol mol^−1^), SAN - soil available N (mg N kg^−1^ soil), and SAP - soil available P (mg P kg^−1^ soil). The abbreviations apply to figures hereafter.

*Significant at *P* ≤ 0.05; **Significant at *P* ≤ 0.01; and ***Significant at *P* ≤ 0.001.
