# Supplementary material for: Soil microbial C:N ratio is a robust indicator of soil productivity for paddy fields
Source: Sci Rep. 2016 Oct 14;6:35266. doi: 10.1038/srep35266 (PMC5064311; doi:10.1038/srep35266)
Supplement: Supplementary Information [file srep35266-s1.doc]

**Soil microbial C:N ratio is a robust indicator of soil productivity for paddy fields**

Yong Li ^1,2*^, Jinshui Wu ^1^, Jianlin Shen ^1^, Shoulong Liu ^1^, Cong Wang ^1^, Dan Chen ^1^, Tieping Huang ^3^ and Jiabao Zhang ^4^

^1^ Changsha Research Station for Agricultural & Environmental Monitoring and

Key Laboratory of Agro-ecological Processes in Subtropical Regions,

Institute of Subtropical Agriculture, Chinese Academy of Sciences,

Hunan 410125, China

^2^ Faculty of Veterinary and Agricultural Sciences, The University of Melbourne, Victoria 3010, Australia

^3^ Hunan Soil and Fertiliser Station, Hunan 41005, China

^4^ Institute of Soil Science, Chinese Academy of Sciences,

Nanjing, Jiangsu 20008, China

**Supplementary Information**

This supplementary information contains 6 tables and 1 figure describing two datasets of paddy fertilisation experiments, one long-term (1987-1999) and one short-term (2012-2014), carried out in rice paddies in Hunan Province, China.

**Table S1. Climate, cropping system and basic soil properties of 0-20 cm at the eight county sites for the long-term dataset.**

| Site | MAT  (^o^C) | Coordinate | MAP  (mm) | Crop rotation | Soil type | pH | SOC  g C kg^-1^ | TSN  g N kg^-1^ | TSP  g P kg^-1^ |
| --- | --- | --- | --- | --- | --- | --- | --- | --- | --- |
| CS | 16.5 | 113°12´E, 28°18´N | 1364 | Rice-rice-wheat | Fe-accumuli-Stagnic Anthrosols | 6.52 | 13.0 | 1.29 | 0.56 |
| HS | 16.9 | 111°45´E, 28°45´N | 1504 | Rice-rice-green manure | Fe-accumuli-Stagnic Anthrosols | 5.65 | 17.3 | 2.01 | 0.42 |
| LL | 16.6 | 111°35´E, 29°32´N | 1314 | Rice-rice-green manure | Fe-accumuli-Stagnic Anthrosols | 5.65 | 20.0 | 2.24 | 0.55 |
| NA | 16.9 | 112°47´E, 29°12´N | 1340 | Rice-rice-rape | Fe-accumuli-Stagnic Anthrosols | 8.05 | 26.4 | 2.72 | 0.81 |
| NX | 17.2 | 112°18´E, 28°07´N | 1553 | Rice-rice-wheat | Fe-accumuli-Stagnic Anthrosols | 5.20 | 17.1 | 1.76 | 0.56 |
| WG | 16.7 | 110°54´E, 26°48´N | 1419 | Rice-rice-green manure | Gleyi-Stagnic Anthrosols | 7.78 | 23.2 | 2.51 | 0.59 |
| XH | 17.0 | 111°42´E, 27°56´N | 1568 | Rice-rice-green manure | Fe-accumuli-Stagnic Anthrosols | 5.20 | 11.3 | 0.92 | 0.60 |
| ZZ | 17.6 | 113°26´E, 27°32´N | 1525 | Rice-rice-wheat | Gleyi-Stagnic Anthrosols | 6.25 | 26.9 | 2.42 | 0.66 |

Note: MAT - mean annual air temperature, MAP - mean annual precipitation, pH - soil pH, SOC - soil organic carbon, TSN - total soil nitrogen, and TSP - total soil phosphorus. The soils at the experimental sites are classified as Stagnic Anthrosols in Chinese soil taxonomy^1^.

**Table S2. Annual application rates (kg ha^-1^ yr^-1^) of N, P and K fertilisers for the five treatments at the eight county sites for the long-term dataset.**

| Site |  | CK |  |  |  | NPK |  |  |  | OM3 |  |  |  | OM6 |  |  |  | CON |  |
| --- | --- | --- | --- | --- | --- | --- | --- | --- | --- | --- | --- | --- | --- | --- | --- | --- | --- | --- | --- |
|  | N | P | K |  | N | P | K |  | N | P | K |  | N | P | K |  | N | P | K |
| CS | 0 | 0 | 0 |  | 540 | 65 | 314 |  | 540 | 73 | 310 |  | 540 | 93 | 290 |  | 513 | 61 | 135 |
| HS | 0 | 0 | 0 |  | 300 | 24 | 140 |  | 300 | 27 | 140 |  | 300 | 33 | 145 |  | 288 | 19 | 100 |
| LL | 0 | 0 | 0 |  | 310 | 45 | 135 |  | 310 | 61 | 177 |  | 310 | 90 | 222 |  | 339 | 52 | 78 |
| NA | 0 | 0 | 0 |  | 510 | 51 | 130 |  | 510 | 51 | 130 |  | 510 | 51 | 130 |  | 510 | 51 | 130 |
| NX | 0 | 0 | 0 |  | 530 | 30 | 218 |  | 530 | 115 | 310 |  | 530 | 208 | 385 |  | 528 | 29 | 174 |
| WG | 0 | 0 | 0 |  | 320 | 38 | 136 |  | 320 | 32 | 137 |  | 320 | 43 | 148 |  | 384 | 46 | 261 |
| XH | 0 | 0 | 0 |  | 315 | 24 | 178 |  | 315 | 24 | 173 |  | 315 | 40 | 176 |  | 357 | 55 | 91 |
| ZZ | 0 | 0 | 0 |  | 540 | 52 | 228 |  | 540 | 78 | 266 |  | 540 | 100 | 308 |  | 548 | 59 | 185 |

**Table S3. Average annual rice yields (mean ± standard deviation, Mg ha^-1^) for 1987-1999 for the five fertilisation treatments at the eight county sites for the long-term dataset.**

| Site | CK | NPK | OM3 | OM6 | CON |
| --- | --- | --- | --- | --- | --- |
| CS | 5.39±0.88 | 10.79±0.89 | 10.96±0.90 | 10.98±0.85 | 10.05±1.22 |
| HS | 6.21±1.07 | 10.45±1.26 | 11.03±1.37 | 10.47±1.32 | 10.19±1.43 |
| LL | 5.80±1.13 | 10.94±0.98 | 12.61±1.35 | 12.29±1.38 | 9.96±0.66 |
| NA | 4.38±0.98 | 9.56±1.02 | 9.60±0.98 | 9.52±0.69 | 9.46±1.02 |
| NX | 6.05±1.10 | 11.43±1.30 | 12.21±2.62 | 12.07±1.46 | 11.20±1.29 |
| WG | 4.86±0.65 | 9.61±0.67 | 10.55±0.68 | 10.87±0.68 | 11.23±1.02 |
| XH | 6.25±0.89 | 12.11±1.04 | 12.37±0.75 | 12.03±0.56 | 11.56±0.60 |
| ZZ | 6.88±1.10 | 10.66±1.01 | 11.98±1.00 | 12.15±0.88 | 10.65±0.72 |

**Table S4. Soil nutrient contents measured after late rice harvest at the eight county sites in 2001 for the long-term dataset.**

| Site | Treatment | SOC  g C kg^-1^ | TSN  g N kg^-1^ | TSP  g P kg^-1^ | SAN  mg N kg^-1^ | SAP  mg P kg^-1^ | SMB C  mg C kg^-1^ | SMB N  mg N kg^-1^ | SMB P  mg P kg^-1^ |
| --- | --- | --- | --- | --- | --- | --- | --- | --- | --- |
| CS | CK | 11.46 | 1.17 | 0.484 | 66.7 | 5.78 | 369 | 37.8 | 5.93 |
| HS | CK | 17.69 | 1.78 | 0.393 | 115.0 | 3.71 | 537 | 33.8 | 6.85 |
| LL | CK | 17.32 | 1.84 | 0.421 | 117.4 | 4.11 | 793 | 83.7 | 13.98 |
| NA | CK | 31.09 | 2.90 | 0.797 | 143.2 | 6.73 | 947 | 106.1 | 23.77 |
| NX | CK | 18.26 | 1.51 | 0.472 | 90.5 | 9.48 | 709 | 70.0 | 13.60 |
| WG | CK | 26.98 | 2.57 | 0.528 | 134.3 | 4.71 | 737 | 79.9 | 17.11 |
| XH | CK | 16.81 | 1.07 | 0.456 | 54.1 | 7.13 | 310 | 33.0 | 8.31 |
| ZZ | CK | 25.69 | 2.06 | 0.412 | 127.3 | 4.38 | 434 | 33.8 | 6.02 |
| CS | NPK | 12.73 | 1.27 | 0.621 | 72.7 | 14.41 | 457 | 42.7 | 6.87 |
| HS | NPK | 19.56 | 1.85 | 0.406 | 125.7 | 5.92 | 705 | 42.5 | 8.16 |
| LL | NPK | 18.90 | 1.83 | 0.692 | 127.9 | 17.95 | 858 | 92.3 | 16.63 |
| NA | NPK | 36.10 | 3.39 | 0.981 | 178.7 | 12.49 | 1144 | 134.0 | 30.48 |
| NX | NPK | 17.76 | 1.73 | 0.548 | 94.7 | 9.01 | 713 | 75.9 | 13.62 |
| WG | NPK | 28.39 | 2.76 | 0.619 | 135.9 | 7.97 | 921 | 122.4 | 17.24 |
| XH | NPK | 16.80 | 1.09 | 0.381 | 41.3 | 5.89 | 319 | 36.1 | 10.67 |
| ZZ | NPK | 25.92 | 2.05 | 0.571 | 126.7 | 7.16 | 459 | 36.3 | 6.30 |
| CS | OM3 | 14.75 | 1.48 | 0.606 | 100.8 | 12.10 | 586 | 58.1 | 8.08 |
| HS | OM3 | 21.10 | 1.95 | 0.423 | 123.8 | 4.31 | 644 | 41.4 | 6.84 |
| LL | OM3 | 20.57 | 1.95 | 0.674 | 136.2 | 15.95 | 1123 | 107.7 | 23.36 |
| NA | OM3 | 34.46 | 3.28 | 0.994 | 165.8 | 11.60 | 1013 | 112.4 | 26.25 |
| NX | OM3 | 24.38 | 2.32 | 0.944 | 134.9 | 56.65 | 1063 | 114.3 | 24.89 |
| WG | OM3 | 35.52 | 3.50 | 0.664 | 167.8 | 8.53 | 1081 | 178.1 | 22.96 |
| XH | OM3 | 19.32 | 1.21 | 0.466 | 55.6 | 6.06 | 392 | 43.0 | 10.24 |
| ZZ | OM3 | 28.86 | 2.18 | 0.784 | 131.5 | 30.02 | 566 | 38.8 | 9.28 |
| CS | OM6 | 16.85 | 1.78 | 0.731 | 105.3 | 18.13 | 736 | 74.2 | 10.75 |
| HS | OM6 | 19.91 | 1.97 | 0.403 | 162.6 | 5.82 | 740 | 43.3 | 8.74 |
| LL | OM6 | 22.17 | 2.06 | 0.701 | 141.5 | 18.74 | 1214 | 99.3 | 21.08 |
| NA | OM6 | 32.32 | 3.10 | 1.046 | 167.4 | 27.39 | 893 | 104.1 | 21.70 |
| NX | OM6 | 27.41 | 2.46 | 1.176 | 174.5 | 81.54 | 1213 | 130.3 | 28.64 |
| WG | OM6 | 38.97 | 3.63 | 0.676 | 181.7 | 8.56 | 1273 | 192.8 | 28.37 |
| XH | OM6 | 22.22 | 1.28 | 0.499 | 65.1 | 7.25 | 419 | 42.5 | 10.30 |
| ZZ | OM6 | 31.50 | 2.25 | 0.847 | 130.9 | 49.58 | 566 | 46.4 | 9.89 |
| CS | CON | 14.82 | 1.42 | 0.56 | 81.5 | 11.64 | 555 | 44.4 | 10.88 |
| HS | CON | 18.41 | 1.81 | 0.503 | 117.3 | 5.75 | 673 | 44.1 | 7.10 |
| LL | CON | 18.62 | 1.95 | 0.567 | 137.3 | 9.32 | 1066 | 104.7 | 20.77 |
| NA | CON | 31.00 | 2.98 | 1.004 | 161.3 | 18.46 | 994 | 131.4 | 26.97 |
| NX | CON | 17.38 | 1.62 | 0.542 | 101.7 | 9.23 | 717 | 73.9 | 17.40 |
| WG | CON | 38.18 | 3.50 | 0.718 | 182.8 | 10.90 | 1238 | 146.3 | 23.75 |
| XH | CON | 16.85 | 1.05 | 0.583 | 44.9 | 17.82 | 304 | 34.7 | 9.08 |
| ZZ | CON | 27.20 | 2.09 | 0.579 | 126.4 | 10.60 | 469 | 35.0 | 6.16 |

Note: SOC - soil organic carbon, TSN - total soil nitrogen, TSP - total soil phosphorus, SAN - soil available nitrogen, SAP - soil available phosphorus, SMB C - soil microbial biomass carbon, SMB N - soil microbial biomass nitrogen, and SMB P - soil microbial biomass phosphorus.

**Table S5. Climate, cropping system and basic soil properties of 0-20 cm at Jinjing in Changsha County for the short-term dataset.**

| Site | MAT  (^o^C) | MAP  (mm) | Crop rotation | pH | SOC  g C kg^-1^ | TSN  g N kg^-1^ | TSP  g P kg^-1^ |
| --- | --- | --- | --- | --- | --- | --- | --- |
| Jinjing,  Changsha County,  Hunan Province | 17.5 | 1,330 | Rice-Rice-Fallow | 5.12 | 18.9 | 2.10 | 0.390 |

Note: MAT - mean annual air temperature, MAP - mean annual precipitation, pH - soil pH value, SOC - soil organic carbon, TSN - total soil nitrogen, and TSP - total soil phosphorus.

**Table S6. Average annual rice yields and soil microbial variables SMB C, SMB N and SMB C:N ratio (mean ± standard deviation) for 2012-2014 for the eight treatments at Jinjing in Changsha County for the short-term dataset.**

| Treatment | Grain yield (Mg ha^-1^) | SMB C (mg C kg^-1^) | SMB N (mg N kg^-1^) | SMB C:N ratio |
| --- | --- | --- | --- | --- |
| NPK | 12.6±0.74 | 632±123 | 51.9±17.6 | 15.6±3.47 |
| NPK+LS | 11.6±1.13 | 710±148 | 53.7±17.2 | 16.8±4.14 |
| NPK+HS | 10.9±1.28 | 772+167 | 57.0±20.3 | 17.5±3.86 |
| NPK+LC | 13.1±1.05 | 719±151 | 60.5±21.5 | 15.0±3.30 |
| NPK+HC | 13.2±0.86 | 673±137 | 57.0±20.4 | 14.9±3.28 |
| 0.5NPK+PM | 11.8±0.75 | 743±143 | 55.7±16.1 | 16.7±4.29 |
| NPK+F | 12.8±1.06 | 555±99 | 45.0±15.6 | 15.2±3.25 |
| NPK+HS+F | 11.6±1.68 | 721±160 | 53.7±21.9 | 16.7±3.73 |


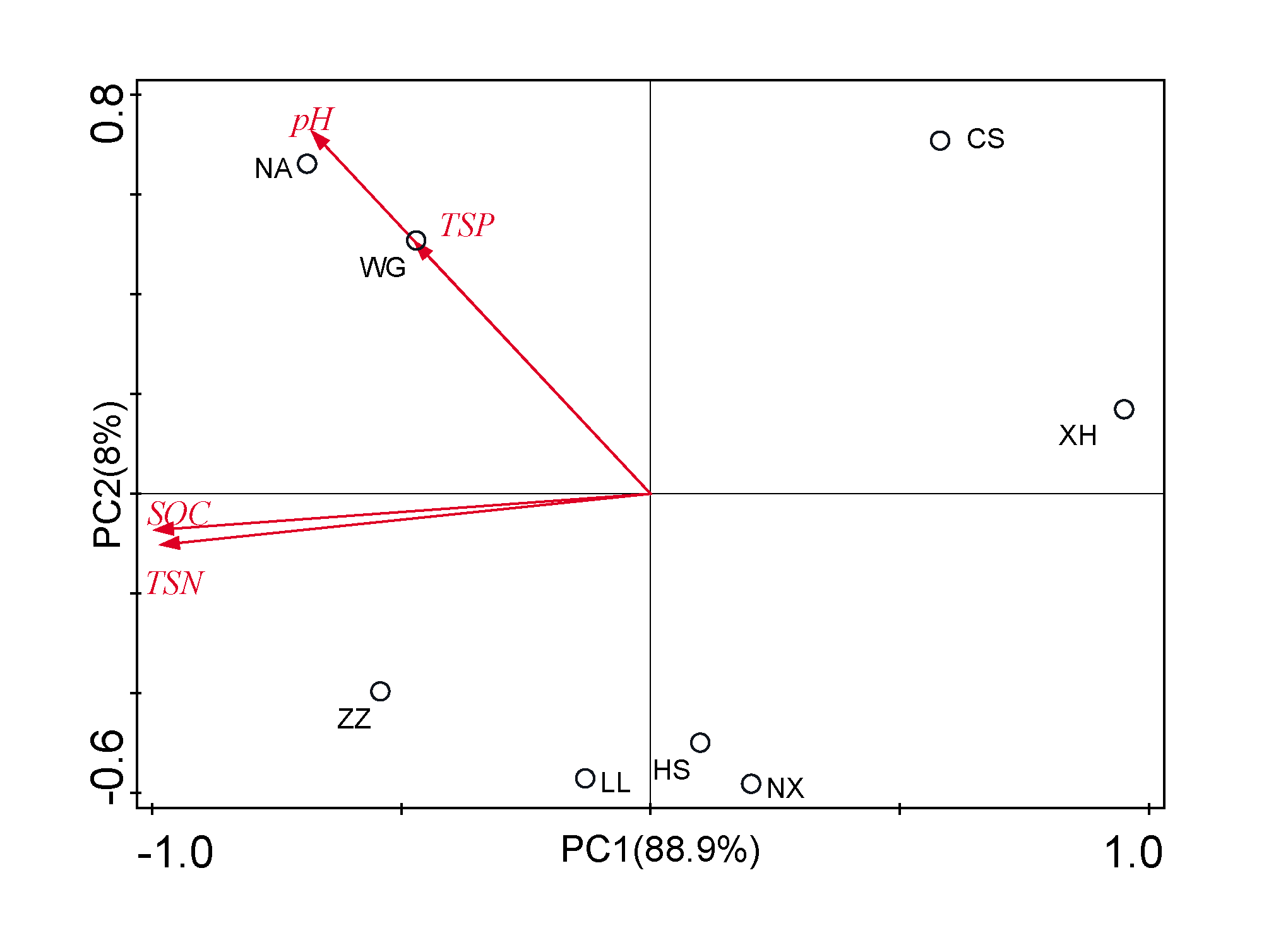


**Figure S1.** Ordination plot of the experimental sites based on soil chemical properties in Table S1 using the principal component analysis for the long-term dataset. SOC, soil organic carbon; TSN, total soil nitrogen; TSP, total soil phosphorus; pH, soil pH. The open circles show the experimental sites (CS - Changsha, HS - Hanshou, LL - Linli, NA - Nanxian, NX - Ningxiang, WG - Wugang, XH – Xinhua, and ZZ - Zhuzhou).

**References**

1. Gong, Z. T., Zhang, G. L., Chen, Z. C., editors. Pedogenesis and soil taxonomy. pp 148 (Science Press, Beijing, 2009).
